# News and Miscellany

**Published:** 1903-02

**Authors:** 


### News and Miscellany.

EXAMINATIONS of urine, sputum, blood, pathological specimens, etc., made at reasonable rates by New Orleans Clinical Laboratory, 124 Baronne Street, New Orleans. O L. Pothier, M. D., Charity Hospital. I. I. Lemann, M. D., Secretary. J. B. Guthrie, M. D.

NEW ORLEANS POLYCLINIC.—Sixteenth annual session opens November 3, 1902, and closes May 3, 1903. Physicians will find the Polyclinic an excellent means for posting themselves upon modern progress in all branches of medicine and surgery. The specialties are fully taught, including laboratory work. For further information address New Orleans Polyclinic, Post Office Box 797, New Orleans, La.

THE ENNO SANDER PRIZE, of the Association of Military Surgeons of the United States for 1903, will be awarded to the author of the best essay on "The Differential Diagnosis of Typhoid Fever in its Earliest States." The board of award will consist of Dr. Austin Flint, of New York; Colonel Calvin DeWitt, of the Army, and Prof. Victor C. Vaughan, of Ann Arbor. Full information concerning the contest may be obtained from Major Evelyn Pilcher, Carlisle, Pa., the Secretary of the Association.

W. B. SAUNDERS & COMPANY desire to announce to the profession that they have established a branch of their business in New York. For this purpose they have secured a suite of rooms in the Fuller Building, Fifth Avenue and Twenty-second Street, centrally located and easily accessible from all parts of the city. Dr. Reed B. Granger, for many years managing editor of the *New York Medical Journal,* together with a representative who is thoroughly familiar with the methods of the Philadelphia house, will be connected with this new branch; and Mr. W. B. Saunders personally will divide his time between New York and Philadelphia.

